# Facilitating leaders to create workplaces where healthcare staff want to work: evaluating a leadership development program

**DOI:** 10.3389/frhs.2026.1768311

**Published:** 2026-07-09

**Authors:** Shaun Cardiff, Donna Michelle Frost

**Affiliations:** People & Healthcare Studies, Fontys Hogescholen, Eindhoven, The Netherlands

**Keywords:** case narrative, leadership development, person-centred leadership, transformational leadership, workforce retention

## Abstract

**Introduction:**

In today's climate of increasing health and social care needs and a declining workforce, most organisations and leaders are not only looking for strategies to recruit new staff but also ways of retaining the staff already in service. Retention, with its multiple factors and no single solution, is a wicked problem. Of the multiple factors influencing staff intent to stay, leadership and culture are two major areas where a significant impact can be made. Front-line leaders are in an ideal position for cultivating workplaces where staff want to work.

**Methods:**

As part of a two-nation project exploring the impact of various interventions on staff retention, we developed and evaluated a leadership development programme for front-line leaders in health and social care. Two cohorts followed a 12 month programme inspired by the co-operative inquiry process and adult, person-centred and transformative learning. Participants were facilitated in moving through continuous cycles of: identifying individual or group action, executing and observing the impact of their actions, sharing their observations and reflecting on the meaning of the observations for leadership intended to encourage staff retention. They creatively expressed their development narrative at the end of the programme.

**Results:**

In this paper we share the participant faction (one fictive narrative based on fourteen participant narratives). The narrative reveals how participants started with feelings of fumbling around in darkness but then discovering one's own (and others') colour as conscious attentiveness to the ways they were leading grew. Alongside finding the authentic self, participants came to the realization that you don't have to travel alone and that using critical thinking and creativity they dared surf the dynamic waves of current health and social care oceans.

**Discussion:**

We conclude that leadership development programmes based on adult, transformative and person-centred learning theory creates a learning climate and culture that enables front-line leaders to start to consciously cultivate workplaces where staff want to stay.

## Introduction

Although improved, the World Health Organisation still estimates a shortage of 4.1 million nurses by 2030. The shortage will be mostly felt in low- and middle-income countries, but higher-income countries can expect dramatic falls due to retirement outpacing recruitment (1). In Europe, even with a predicted rise of 8% of qualified nurses by 2071 and constant recruitment and retention, a shortage of nurses is expected because of demands created by an ageing population (2). In the lowlands of Belgium and The Netherlands, this shortage is already evident. In The Netherlands, staff shortages are currently at their highest in the healthcare and social wellbeing sector, with nursing the profession most in demand (3). Since 2015, nursing has been in the top ten professions with a workforce shortage in Belgium, and was number one in 2021–2023 (4). With increasingly complex healthcare needs and our largest healthcare profession consistently classed as a bottleneck profession, retention should be a major priority for all health- and social care organisations.

High turnover and lack of consistency in healthcare staffing has been shown to negatively affect quality of care as well as incur high costs within various sectors throughout the world (5–7). Reducing staff turnover is therefore important and best approached by creating workable work: work that does not make one sick or stressed; that is motivating and interesting and offers opportunities for learning and development. However, creating working environments where staff want to work also requires awareness of the factors influencing staff intention to stay or leave.

**Figure 1:**
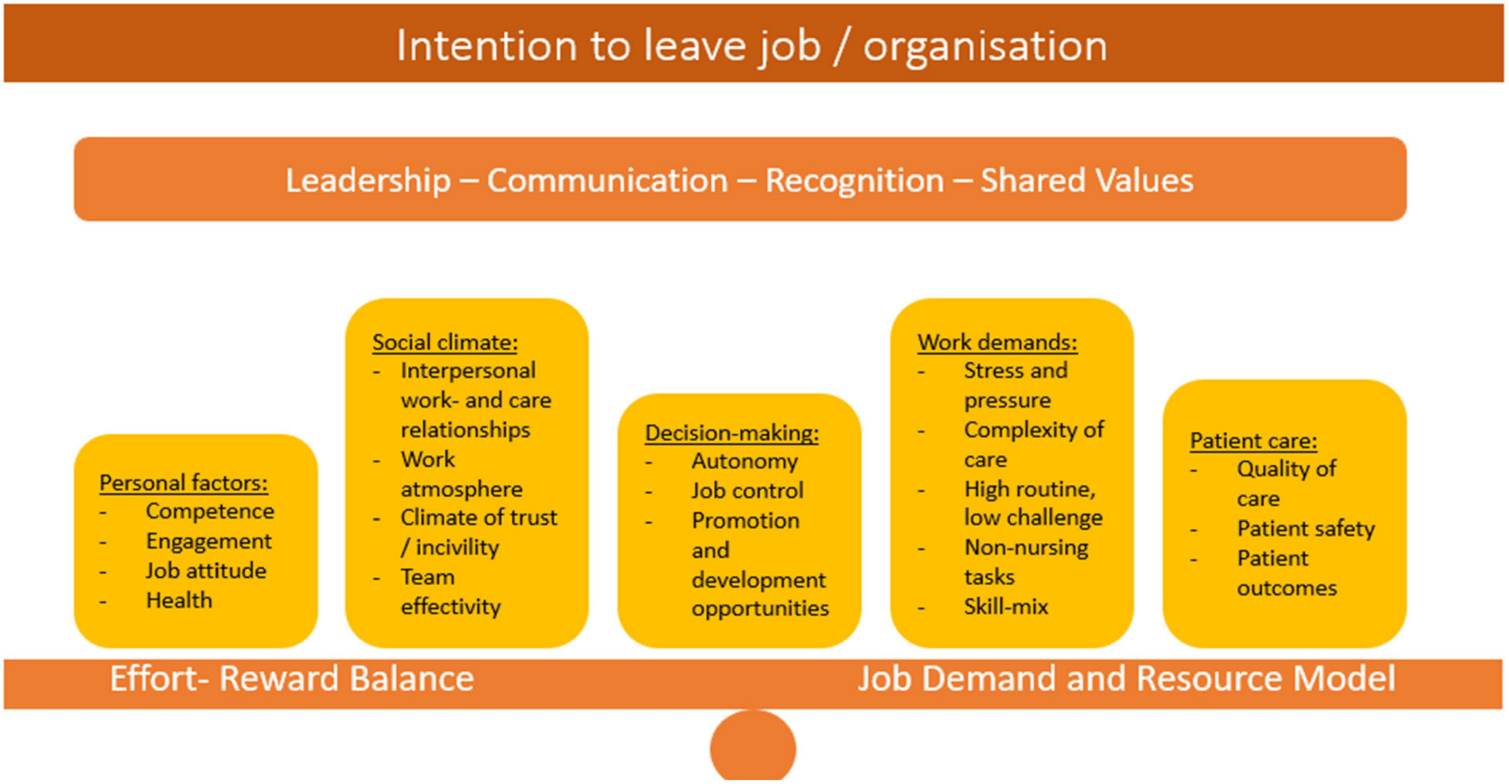
Factors and themes influencing healthcare staff intent to leave their job or organisation (9).

As part of a European pilot study exploring ways of positively influencing staff intent to stay (8), Averens et al. (9) conducted a literature review and identified four overarching factors and five themes influencing healthcare staff intention to leave their job or organisation (see Figure 1). The themes include:

1. Personal factors, including socio-demographic characteristics such as circumstances at home, professional identity, health, wellbeing and professional competency, all of which can influence work engagement and commitment.
2. Social climate at work, created by the interpersonal relationships staff engage in, including relationships with service-users (patients/clients/residents), colleagues and management. Positive relationships and a climate of trust and respect (as opposed to incivility) support a sense of belonging and resilience.
3. Decision-making at work, which concerns those opportunities where staff can influence the decisions made within the workspace, particularly those influencing their working environment and professional development.
4. Work requirements are the job demands that influence staff mental, physical and emotional wellbeing. Work environment characteristics such as insufficient staffing can increase job demands and foster intentions to leave.
5. Quality of care outcomes such as (perceived) service-user safety, satisfaction, progress and care not being left undone, can also influence staff intent to stay.

The four overarching factors include:

1. Respect and recognition from service-users (patients/clients/residents), colleagues, management and society in general.
2. Shared values and a shared vision fostering a sense of belonging, engagement and commitment.
3. Communication which is considered timely and adequate, flowing down through the organizational hierarchy and positively influencing team effectiveness, quality of care and staff satisfaction.
4. Leadership, by higher management and those with whom staff have the most contact, is the last pivotal factor influencing intentions to stay.

The fact that these themes are numerous and interconnected makes retention a “wicked problem” best approached through interventions aimed at fostering staff empowerment (10). After their review of influencing factors, Averens et al. (11) conducted a scoping review of European intervention studies on improved staff satisfaction and reduced intent to leave. Whilst they found a lack of consistent evidence for any one way of improving staff satisfaction or intent to stay, they did find evidence that interventions aimed at improving the psychosocial working conditions of staff were effective in positively influencing staff intent to stay. Their conclusion was that (repeated) interventions aimed at both individuals and the organization had a longer lasting effect.

As a partner in the same European study, Cardiff et al. (12), conducted a realist review and found sufficient evidence to support a tentative program theory that states: when local, first-line nurse leaders focus on developing positive leadership relationships and work environments, nurse retention within the team, organisation and profession are sustained or improved. When front-line nurse leaders focus on fostering relational connectedness with (and among) staff, enable professional practice autonomy, cultivate healthful workplace cultures and facilitate professional growth and development, they positively impact staff intention to stay.

Person-centred theory sees healthful cultures that foster human flourishing among service-users, their significant others and healthcare staff as the outcome of person-centred practice (13). Deuling et al. (14) also recommend focusing on values and cultures when developing person-centred care as the development is as much dependent on organisational structures as on individual leaders. Traditionally, transformational leadership, particularly Kouzes & Posners (15) leadership model, has been the recommended leadership style for transformative practice development (16) and person-centred practice (17). Searching for what fosters a workplace culture where healthcare staff want to work, Cardiff et al. (18) identified collective leadership as a key guiding light, alongside living shared values, creating safe, critical and creative learning environments and focusing on change that font-line staff and service-users consider “good for all”. Amin et al. (19), conclude that a person-centred approach to leadership is inclusive and encourages contribution from a range of individuals and groups, capitalising on their strengths for the benefit of all.

**Figure 2:**
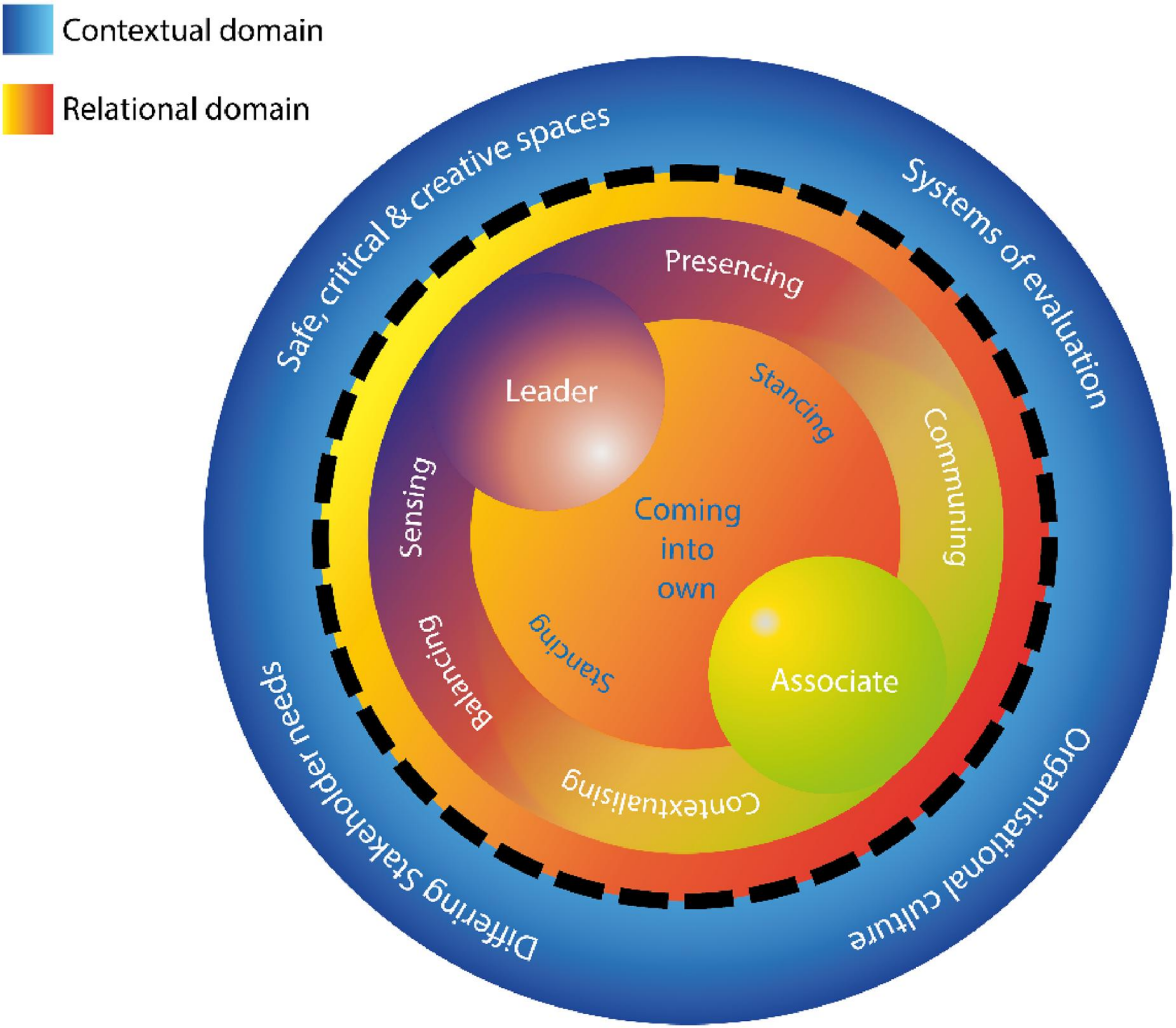
A conceptual framework for person-centred leadership (22).

The COVID-19 pandemic really highlighted the importance of a more relational approach to hierarchical leadership for enabling and maintaining healthful workplace cultures as well as how organisational environment can support or inhibit such leadership (20). Coming out of the pandemic, Dickson et al's (21) rapid realist review found that healthful leadership strategies include: being visible and present; open and engaging; caring for self and others; embodying values; prepared and preparing others, and using available information and support. Such studies support Cardiff et al's (22) conceptual framework for person-centred leadership (see Figure 2) which is defined as a complex, dynamic, relational and contextualised practice aiming to enable associates and leaders achieve self-actualisation, empowerment and well-being. The leader utilises attributes of authentic caring and other-centredness, intra- and interpersonal intelligence, patience, optimism, openness, a willingness to show their own vulnerability and reflexivity, to engage effectively in relational processes (sensing, presencing, communing, balancing needs and contextualisation) and continuously (re-)position themselves in relation to those they are leading and so enable the coming into own of others and self. This, whilst working with and under the influence of surrounding contextual factors.

To summarize, this section argues that the retention of healthcare professionals is a wicked problem and in crisis. Leadership is a major factor influencing staff intent to stay within their job or organization, and frontline hierarchical leaders can have a positive influence when they focus on fostering relational connectedness (feeling safe, respected and belonging), staff autonomy, development and a healthful workplace culture. Healthful cultures are the outcome of person-centred practice, and due to their alignment of core values and principles, transformational and person-centred leadership would be a logical choice of leadership theory and style for leadership development programs.

This paper presents the developmental journey of participants on a leadership development program delivered as part of a European study aimed at addressing the staff retention issue. The program design addresses an observed praxis gap in leadership development programming i.e., how participant leaders can practically apply theories, beliefs and skills with moral intent in their practice, rather than just understand the theory itself. Traditionally, leadership development initiatives have a predetermined program of theories and competencies that developers feel participants should be applying to their contexts. Bodrick e.a (23) make a strong argument that such adult learning should be more learner-centred, inclusive and participatory with co-transforming and co-innovating methods. We therefore developed a program grounded in reciprocal and discovery-based approaches that are aligned with person-centered and adult transformative learning theory. Rather than delivering a predetermined content-focused program teaching leadership behaviors we felt participants should learn, our program structure focused on facilitating cooperative inquiry process that foster the embodying (authentically seeing and doing) person-centred and transformative ways of leading, and embedding them within the practice environment. The intent is for leaders to discover what they need to develop and grow in their specific contexts, despite systemic constraints. The results section of this paper offers insight into the developmental journey from the participant perspective, and the discussion section focuses more on the learning and transformative processes than knowledge retention or behavioral compliance.

## The “Leading to Connect and Retain” development program

Leadership development programs are known to be beneficial to organisations and individuals, but also time consuming and costly. Recigno et al. (24) describe leadership development as a process that takes time and conscious effort as well as being a destination. It is achieved via learning strategies such as continuous (self-)reflection and activities such as role play, journal clubs, shadowing etc, alongside use of leadership frameworks to analyse and guide meaning-making and action-taking. Socialization and mentorship also expose leaders to the influence and support of others and so contribute to leader development, as does the facilitation of leadership development in others. Lastly, a supportive work environment and educational programs (with fieldwork) will support leadership development. Seidman et al. (25) identified 4 potential organisational benefits of leadership development programs: benefits to other staff such as feeling respected, supported and enabled to act in collaborative problem solving and teamwork, whereby reduced leader intervention is needed and general workplace morale enhanced; improved patient safety and satisfaction; tangible project benefits of those projects that were part of the programme, and self-reported improved leadership competency.

In 2021 the first author of this paper was involved in a pilot project examining the question of how to reduce healthcare staff turnover in the European lowlands of Belgium and The Netherlands (8). One output of that project was a leadership development programme specifically designed for front-line hierarchical leaders such as charge nurses/ward managers and team leaders: the Leading to Connect and Retain (LCR) program. This program was further refined in the successional project, reported in this paper, which focused on ways of improving staff retention as well as adaptive and innovative capacity within health- and social care organisations (26).

**Figure 3:**
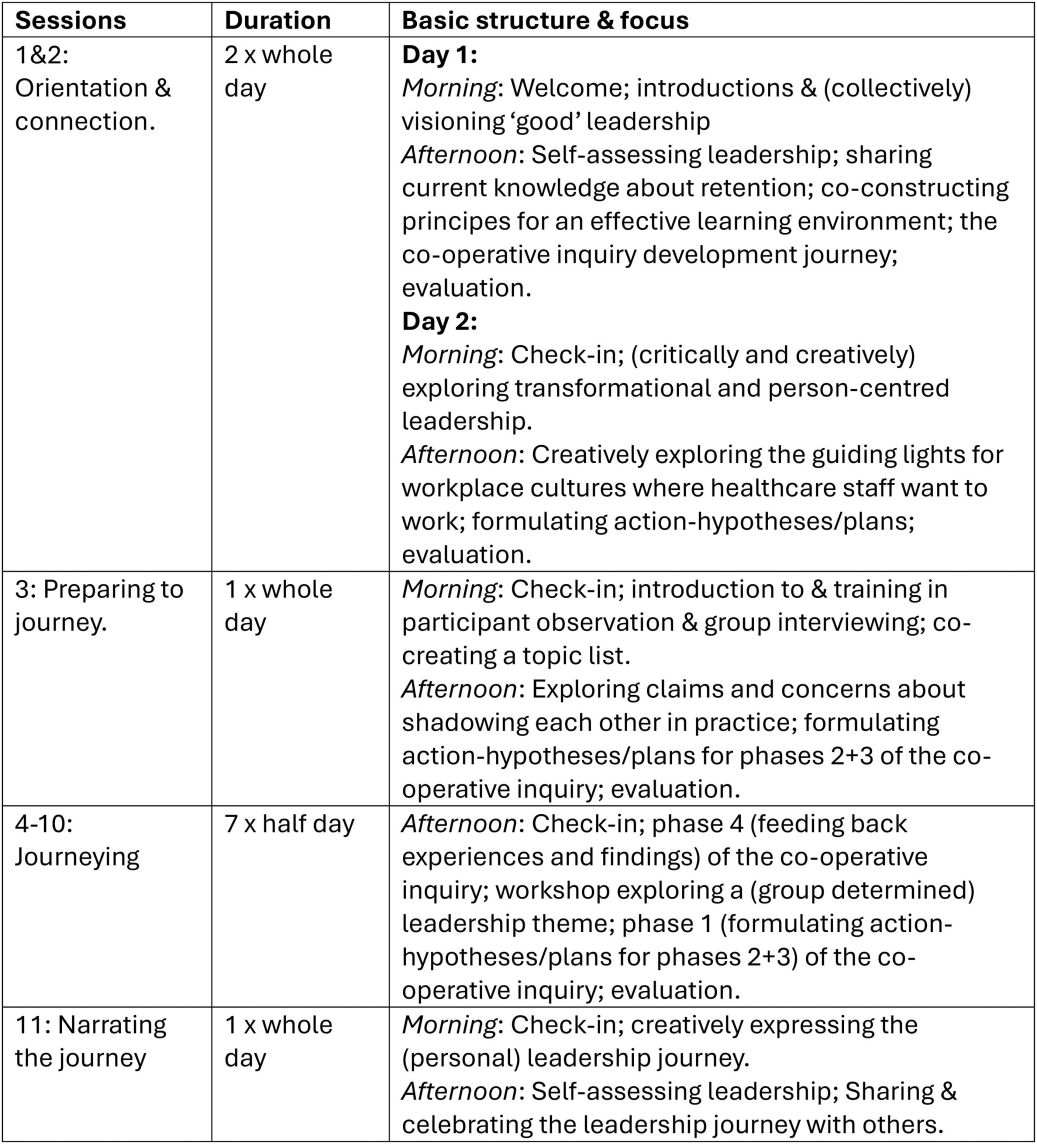
The “Leading for Connection and Retention” program: basic structure.

The LCR program design and delivery was inspired by the methodological and pedagogical principles of a person-centred curriculum (27). It was designed to: be transformative and enable journeying through knowing, doing, being and becoming a more transformative and person-centred leader; be co-constructed, flexible and adaptive to the learner; encourage connectivity with self, other persons and contexts, and be built on a philosophy of pragmatism. Details of the basic design are depicted in Figure 3.

Day one of the program focuses on participant connectivity with each other, as well as with personal and shared visions of good leadership. Day two introduces the theoretical frameworks of transformational (15) and person-centred leadership (22). Day three focuses on equipping participants to conduct site visits to shadow a fellow participant within the workplace for a day, and conduct a group interview with the leader's team. In line with the person-centred principle of respecting a person's right to self-determination, peer shadowing is not compulsory.

Days four to ten are structured to follow a cyclical process of discovery and action planning, inspired by co-operative inquiry. Two experienced researcher-facilitators guide the cohort through the co-operative inquiry process of researching their own practice. Using Heron & Reason's (28) four phased structure, participants are facilitated in formulating individual and collective assumptions about how to foster staff intention-to-stay (phase 1), then translate these into leadership activities which are enacted in practice (phase 2), observed for (staff) responses (phase 3) and the findings fed back to the group at the next meeting for critical dialogue and formulation of new knowledge (phase 4). This then initiates a new cycle in inquiry. During sessions 4–10 there is also an open space for creative workshops. Workshop themes are based on participant needs/desires/requests and co-decided during the previous session so that the program facilitators have time to prepare the workshop delivery.

The LCR program was delivered to two cohorts, averaging one session a month spread across 12 months (per cohort). Twenty-one leaders enrolled for cohort 1 and nineteen for cohort 2. For both cohorts, fifteen participants attended the initial sessions. In cohort 1 five participants dropped out during the program and attendance rates averaged 71% (range: 46%–100%). For cohort 2 four participants dropped out during the program and attendance rates averaged 79% (range: 58%–100%).

The LCR program was one of three developmental programs designed for the European project. The others were: (a) team champion program for team members wanting to foster wellbeing within the team and workplace; (b) a support program for newly qualified nursing staff. Originally, outcomes were to be monitored via a monthly and six monthly survey using multiple validated instruments covering staff outcomes such as intention-to-leave, job satisfaction, empowerment, belongingness, professional competency, leadership and vitality. The surveys were to be collected via an application developed for the study. The app also presented results on dashboards for immediate feedback at an individual and team level. Unfortunately, the application development proved to be so complex that gathering sufficient quantitative data for meaningful results was no longer feasible within the time frame and alternative means of evaluating the programs had to be sought.

The LCR program evaluation strategy included: (a) cohort group interviews conducted by an independent researcher halfway and at the end of the program; (b) leadership self-assessment using a Dutch version of Kouzes & Posner's Leadership Practice Inventory (29), and of the Management Support Scale (30) at the beginning, middle and end of the program; (c) leader development narratives collected during the last session of the program. This manuscript reports specifically on the narrated perspective of LCR participants and answers the evaluation question: what impact does the LCR program have on participants as leaders of self and others in health and social care settings, as related by their developmental narratives? Having collected and analysed personal participant narratives, a case narrative is presented here as a fictive narrative based on the actual narratives of those leaders attending the last session of the LCR program. The methods of data gathering, analysis and case construction are presented in the next section.

## Method

Evaluations of healthcare leadership development programs are diverse in scope and focus. This is due to multiple conceptualisations of leadership underpinning programs, pedagogical principles informing curriculum design and delivery, stakeholder perspectives gathered, outcomes measured and data gathering methods used (31). Explicating the “what” and “why” of evaluations is therefore important.

Leadership, as described in the LCR program, is a relational and moral practice in which a front-line hierarchical leader attempts to exert influence and co-create healthful workplace cultures through self, other individuals and groups within the health or social care organisation. It is best served by person-centred and transformational leadership theory.

Person-centredness is also the core value guiding the design and delivery of the LCR program. We believe it centralises personhood and colours the way we, as facilitators of learning, try to relate with participants so that all (facilitators and learners) strive to think and act from reciprocal respect and understanding whilst trying to co-create a learning environment in which all can come into their own (32). As educators and researchers moving from a critical social science paradigm, we also believe that the intended transformative journey undertaken by participants follows the “consciousness raising—empowerment—emancipation” route described by Brian Fay (33). Inspired by critical creativity theory (34, 35), we also believe in the use of creative methods to enable the explication of embodied knowledge and prevent critical dialogues stagnating in semantic discussions before a sense of shared understanding has materialised. Against this background, we decided to evaluate the impact of the LCR program on participants as leaders of self and others in health and social care settings, by gathering their developmental stories and constructing a case-narrative.

Case narratives are constructed by gathering, blending and melding a variety of participant reflections of their lived experience, then re-presenting them collectively as one (fictional) participant journey. In doing so, the case narrative incorporates the experiences felt and actions, events or happenings observed by participants across time. The interweaving of multiple events and perspectives into a rich, coherent and temporally organised description of the phenomenon of study is aimed at enabling reader understanding of the complexity, contextuality and particularity of the phenomenon (36, 37), in this case the LCR participant leadership development journey.

### Participants

All fourteen participants attending the last session of each LCR program (seven in cohort 1; seven in cohort 2) shared their personal leadership development journey. Of the thirteen females and one male participant, four worked in hospitals and the remaining leaders worked in (long term) residential or community care. Professional backgrounds included nursing, allied health and social work.

### Data gathering

The case narrative presented in the results section of this paper is a blending and melding of fourteen creatively expressed leader narratives collected in a four phased workshop during the last day of the LCR program. The four phases are:

1. *Contemplation*: Participants were invited to first spend 15 min engaging in quiet contemplation of their developmental journey. This could be done by sitting quietly in a secluded area of the room, or a contemplative walk within the building and surrounding grounds.
2. *Creative expression*: Returning to the central space, participants were invited to use the image cards, arts and crafts materials displayed across several tables, to creatively express their journey on an A2-sized flipchart. We reassured them that aesthetics was not of importance and encouraged them to act intuitively first, collecting imagery and materials which drew their attention before thinking about how to use them meaningfully. Working in silence was encouraged and we consciously tried to create a space with minimal distractions or disturbances. Most participants completed their collage within 45 min, and we only moved on to the next phase when it was evident that each participant was ready to move on.
3. *Sharing*: Moving around the room from one collage to the next, each person was asked to share their leader development journey whilst others listened attentively. A photo was taken of their creative expression and an audio-recording of their story. When it appeared like the person had shared what they wanted to share, we posed one question: “Is there anything else you would like to share about your journey?” More often than not, this would initiate further elaboration, enriching the initial story. The narrator was then asked if they would like to hear any reactions from the listening public, and if so, other participants were invited to share their thoughts in supportive ways. As facilitators of such processes, we would often role model supportive reactions using phrases such as: “I see … I feel …. This reminds me of …” If the sharing of the personal journey was emotional for the participant, we were conscious of being sympathetically present. This often just entailed giving them question-free space to collect their thoughts and feelings in their own tempo before moving on, or offering them the option of leaving the space as we moved on to the next participant, or for them to move on to the next part of their own story. Only one participant did not feel ready to share her story in the presence of others and choose to share it with one of the facilitators in a private, recorded session.
4. *Transcribing*: The audio-recordings of all stories told were transcribed, identifying details removed or fictionalised, and the transcript reconstructed as a cohesive narrative accompanied by the photo of the creative expression embedded within the text. The narrative was then emailed to each participant for member-check.

### Data analysis

To construct the case narrative the two researcher-facilitators (authors) engaged in a critical and creative hermeneutic analysis (38) of the fourteen participant development stories in order to define a thematic framework. The framework was then used to construct a faction: a case narrative in which the individual stories are amalgamated into an overarching narrative about leadership development. The process was as follows:

1. Familiarization—close to the scheduled analysis meeting, both authors read through all fourteen, member-checked narratives, back-to-back. We then took some quiet contemplation time to reflect on the participant journeys, engage in a critical dialogue with self and form a general understanding of the participant leadership development journey. Initial thoughts were jotted down in a few keywords or visualised using image cards.
2. Grounding and expressing—upon entering the scheduled analysis meeting we took a few minutes to ground ourselves, sitting quietly and allowing the fourteen narratives to drift in and out of our conscious mind. When we felt ready, we repeatedly and individually scanned the image cards already displayed in the room, collecting those cards that caught our attention. Each of us then thoughtfully arranged our own collected cards to reflect our perception of the essence of the fourteen LCR participant narratives.
3. Blending and melding—through critical dialogue with each other we [to use McCormack & Titchen's (35) critical creativity phrases] blended our individual expressions into a new melded synthesis representing the participant journey. This entailed viewing each other's image card collage, listening attentively to each other's explanations, questioning vagueness or inconsistency and collaboratively delineating core themes. Contestation and debate continued until we had consensus for a thematic framework. Data from the original fourteen participant narratives was then mapped onto the thematic framework. No extraneous data was left nor new themes identified.
4. (Re-)Constructing—having been formed through a critical and creative blending and melding of the fourteen narratives during phase 3, the thematic framework and data was used to construct one case narrative: “… an account of an event/activity/process, bound by time and told from the perspective of one or more persons involved” (36: p.2). The case narrative (presented in the results section below) has a basic narrative structure of an introduction (in which the scene is set), a core (one section per theme from the thematic framework, filled with text from the original participant narratives) and a conclusion.

### Ethical considerations

The fourteen participants of this evaluation study entered the LCR program voluntarily and were not obliged to attend or participate in any of the program activities. On day 11, at the beginning of the creative narrative workshop, participants were informed of the purpose of the workshop (creatively narrating their personal leadership development journey) and how the individual stories would be blended and melded into one fictional participant case narrative. Opting out of contributing to the case narrative was possible at all stages between data gathering and member-checking the final version. Personal details such as name, place of work, faces on photographs etcetera were removed from the individual narratives before the data analysis process began.

We would consider the approach used in this study to be aligned to person-centred research (39). Firstly, participants were never considered as merely a means of delivering and evaluating the LCR program. The narrative workshop was itself a method for participant reflection and consciousness raising towards their own leadership development. Participants self-determined both what and how they could fulfil their potential as leaders of others and express their developmental journey at the end of the program. The narrative approach enabled evaluation to start where each person was at and express what mattered to them. As person-centred facilitators and researchers we were reflexive, aware of the power and influence our position brought with it and conscious of creating I-thou relationships through respect of personhood and participant right to self-determination. Creating safe learning environments during the program fostered mutual respect, understanding and reciprocal trust. Consequently, none of the participants present at day 11 opted out of the evaluation process and no adjustments were needed during member-check.

## Results

The following case narrative entitled *My coming out,* describes participant development during the LCR program in five themes and the imagery used to illustrate each themes uses the same image cards used in phase three of the data analysis process.

Starting with feelings of fumbling around in the darkness of professional leadership practice and the first days of the development program, participants journeyed into consciously moving with (one's own and others') colour. It was a journey of discovery, finding the authentic self, but also the realization that you don't have to travel alone in (learning) leading self and others. By using critical thinking and creativity, they dared surf the dynamic waves of the health and social care ocean.

### My coming out

The past year has been a period of growth and development for me. At the beginning, I felt uncertain, as if I were sometimes drifting in a fog, doing more of the same thing over and over again but not making any progress, not always knowing how to approach things differently (Theme 1). Now I know how to take conscious steps to help others discover their true colours and help them work together. It's about listening, seeing people for who they are, recognizing their strengths, and celebrating successes together.

**THEME 1:**
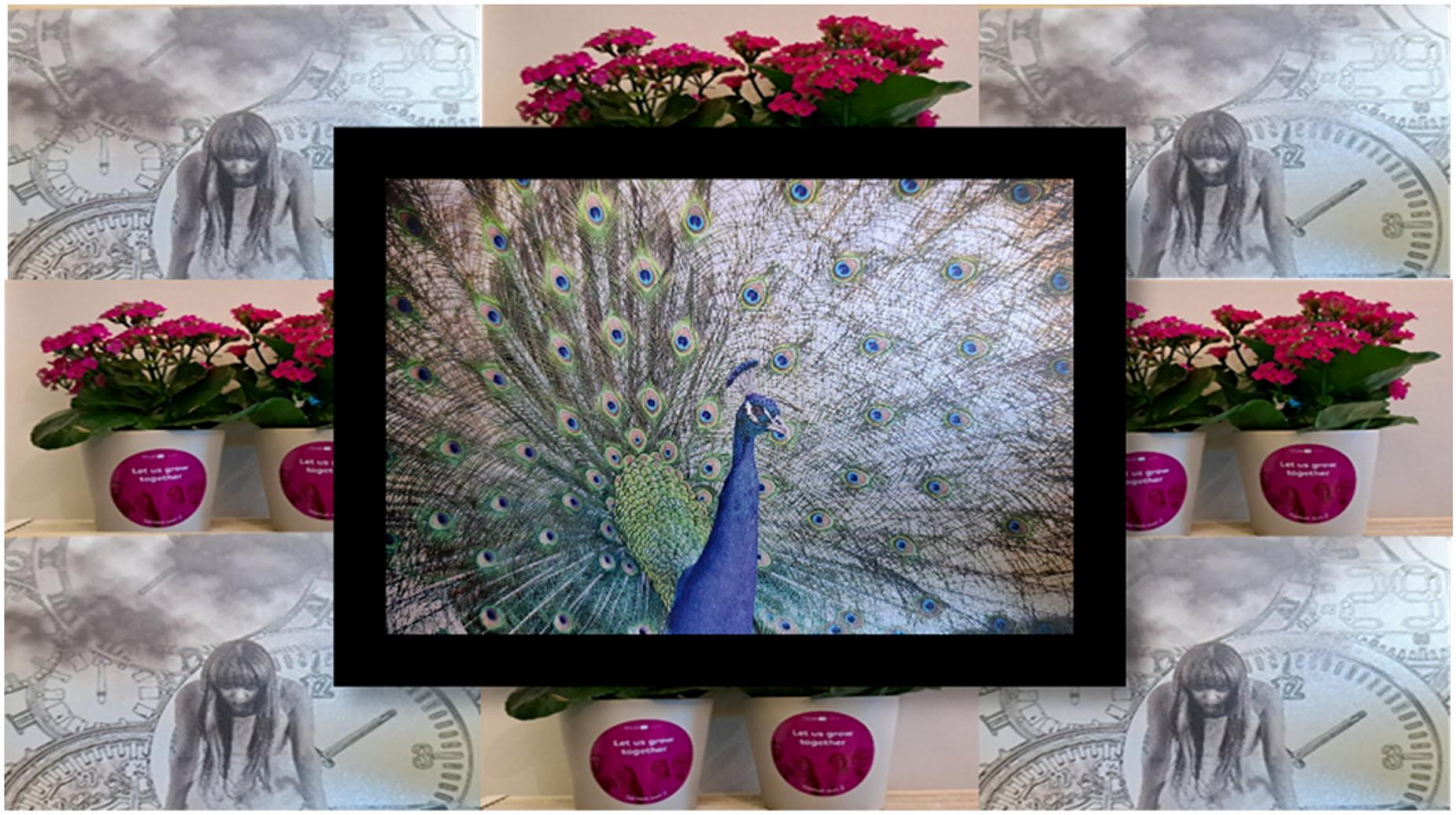
From fumbling around in darkness to consciously moving with colour.

This development wasn't without its challenges. I struggled with limiting beliefs, doubts about my gentleness and capabilities, and sometimes lost sight of the bigger picture. During the process, I learned that everyone has expectations and a background, and that enabling staff participation and listening to each other are essential for shared responsibility. It empowered me to embrace my role as a leader: staying on course, setting boundaries, and discussing difficult issues. Just like in chess, I learned to think ahead, consciously reflect on my choices, and anticipate reactions. This gave me joy in my work and more confidence in my role.

Leadership turned out to be more than leading a team; it's also a way of life. I discovered that what receives attention grows, and that listening and asking questions often yields more than telling someone what to do. And paying attention to both staff and service-users has become a guiding principle. Because only through the staff can we truly be attentive to service-users.

The year felt like a rollercoaster, but I gradually became more confident. I dare to express my ideas, give feedback, and lead by example. The helicopter view—taking off briefly and looking down—also helps me understand situations better and make decisions more calmly. I've become more open, listening to all ideas and looking for useful elements, even if they don't seem immediately realistic. Compliments and appreciation are important in this: bringing lightness, being the cheerleader, but also setting boundaries when necessary.

I've found peace and am feeling grounded. My work-life balance has improved, although I need to be attentive to it. I've learned to compartmentalise my worries and let them go before I go home. In my team, I work to create a safe environment where everyone can be themselves and support each other. I approach projects differently: with more input and involvement. Small things, like deciding together where new computers will be installed, do make a big difference.

I now see my team as a group of individuals with unique qualities and colours. My role is to provide direction, guide them out of the harbour, but also let them find their own way. Sometimes that requires hierarchical decisions, but above all, it's about giving them space and connecting with everyone's strengths. I've learned that there's no single right way, and that as a leader, I sometimes have to adopt a different “colour” too. I've gained a better understanding of the bigger picture.

Initially, I was mainly preoccupied with insecurities, wanting to do everything right and wondering whether I was a good leader. I gradually discovered that leadership starts with being true to who I really am (Theme 2). There's no one ideal leader profile that I have to conform to. I can be myself as a leader and as a person, with my own unique qualities, strengths, and areas for development.

**THEME 2:**
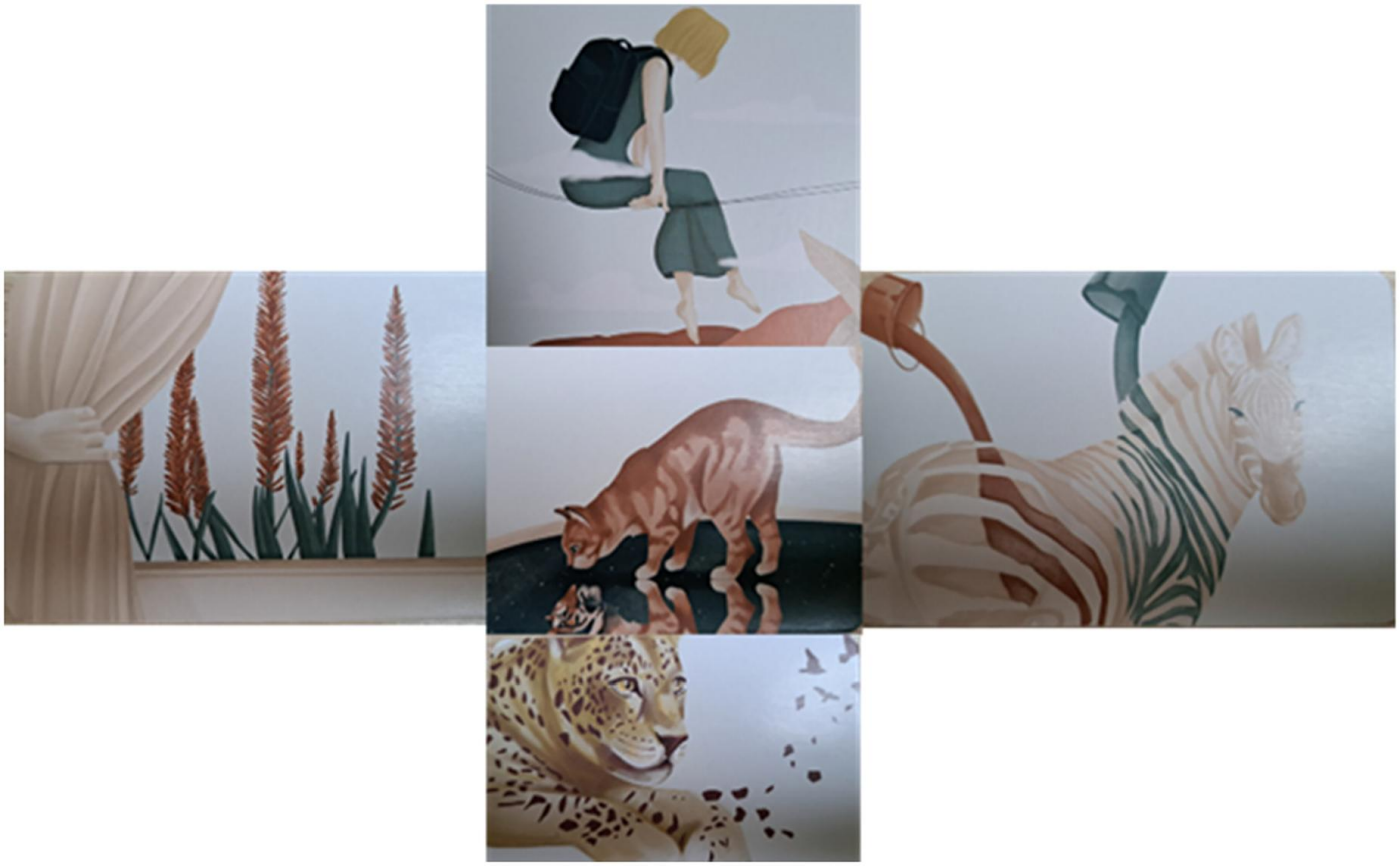
Finding the authentic self.

In the beginning, I often held back, afraid of making mistakes or saying the wrong thing. I sometimes felt like an outsider, trapped in patterns and doubts. I wondered: “Do I belong here?” But through conversations, reflection, and support from others, I've learned that my individuality is actually valuable. Showing personal interest, asking about someone's home life, or giving compliments aren't weaknesses but expressions of my authenticity.

A key insight was that I first had to take good care of myself before I could be there for others. This meant setting boundaries, closing my door at times, and taking time for reflection. Showing vulnerability—to my team, colleagues, and myself—has brought me closer to my own core. By consciously examining myself, I've learned to “test” my actions against my own values. I now dare to acknowledge my insecurities and see that making mistakes is part of the growth. I've become more compassionate to myself and, as a result, with others.

Diversity within the team is valuable; it's not necessary for everyone to be the same. It's precisely the differences that make us stronger. I've also discovered that as a leader, I don't have a fixed position. Sometimes I'm among my team, sometimes beside them, and sometimes above them. And a key turning point for me was making my voice heard in collaboration with other managers or with my own manager. Where I had previously been reluctant, I now realized that my input does matter. That felt like a victory: not just being present, but actively contributing from my own strengths.

My development feels like a transition from winter to spring. I've learned that authentic leadership is about balance: being open and setting boundaries, gentleness and firmness, being myself and adapting. Ultimately, I've achieved being true to my own colour—my authentic self—and that I can lead from that foundation.

I now realize the importance of creating “space” for myself: space for contemplation and reflection; being easily accessible to colleagues while protecting my own space and time; and creating space to be vulnerable. But, I've also learned that I don't have to do it all alone (Theme 3). While I used to be inclined to take on tasks myself and solve everything, I've now learned that leadership is actually about carrying the load together, building and growing together. As a leader, I do have a specific role and specific responsibility, but it's not about solving everything on my own. It's about enabling us all to address the challenges we face together.

**THEME 3:**
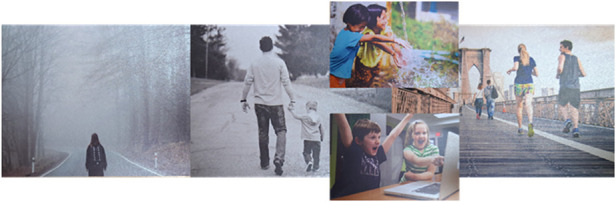
I don't have to be alone.

I've learned that my team members are the stars, each with their own unique qualities and colours. My role is to provide direction and facilitate. This involves brainstorming, using creative working methods, and, above all, engaging in dialogue. I no longer lay all problems at my own feet, I also bring them to the team: “How are we going to solve this?” This is how I foster ownership and shared responsibility.

Previously, by not sharing, I became isolated. In the [*LCR*] group I learned that we're all in the same boat, with shared challenges and successes. Sharing experiences, tips and tricks gave me peace of mind and confirmation that I'm not alone. Now I know I can ask for support—from my team, from fellow managers, and from my director. Realizing that I always have influence, both upwards and downwards, has changed my perspective. It's about transparency, making agreements, and adhering to a shared vision.

Where I used to primarily act based on what I thought I should do, I now have a theoretical framework with four pillars: relational connectedness, a safe workplace culture, fostering growth and professional autonomy. These pillars give me direction and consistency in helping me justify my course of action (Theme 4). They help me approach projects differently and foster greater ownership within the team. No longer just saying “that's just how we do it”, but working together to find what works.

**THEME 4:**
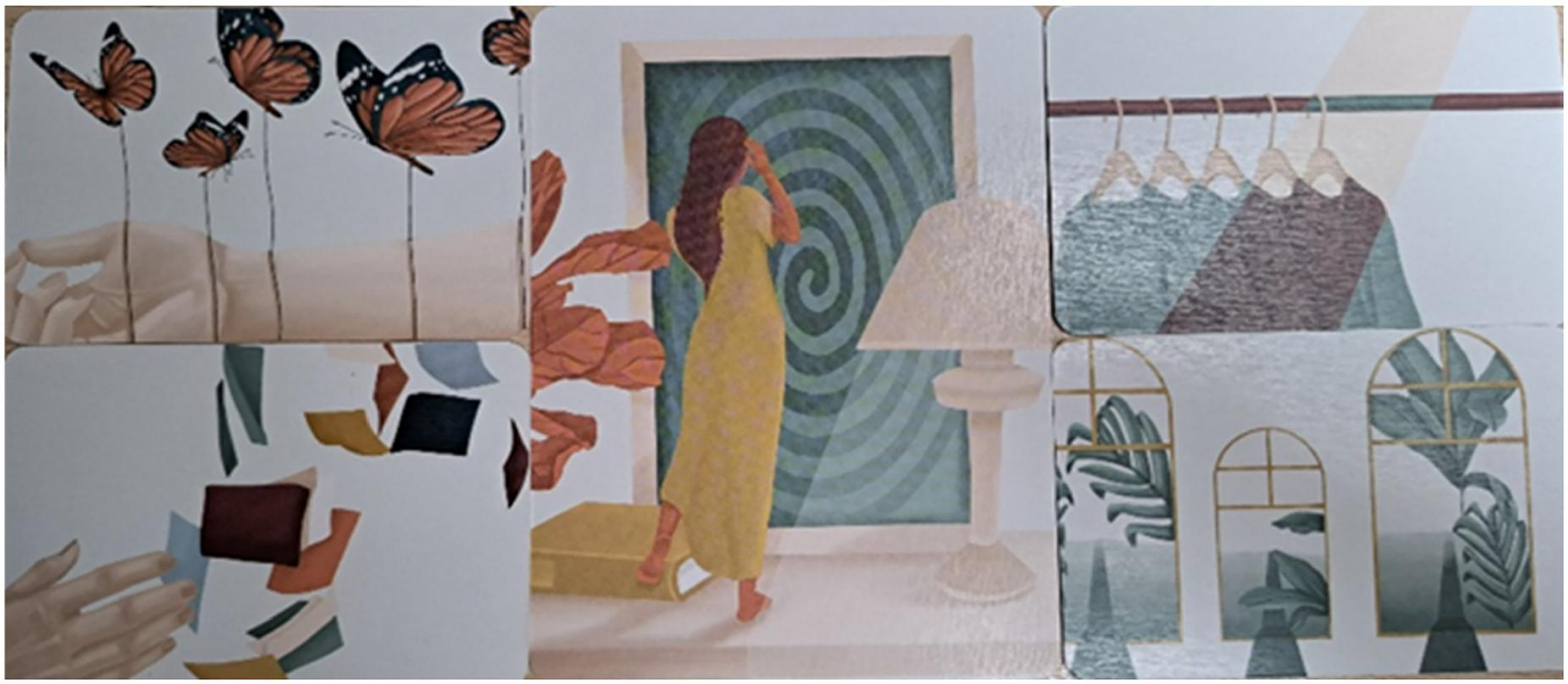
Being critical and creative.

In our collaborative search for what works, I've learned to utilize and value creativity and creative approaches. In conversations and meetings, I use post-its, image cards and creative methods that encourage people to contribute their ideas and feel involved. This creates more depth and energy. Staff respond positively, feel seen and are confident in contributing their own ideas. Using metaphors, such as the tugboat guiding a large freighter, helped me understand and explain my role: to provide direction, connect and guide the team, but, also let go so they can navigate their own path. Creativity helped me not only find a single path towards the end goal, but also explore side roads.

Besides creativity, I learned to be more critical. The facilitators held up a mirror to me and asked questions that forced me to think more concretely and express my thoughts more clearly. This provided new insights, and I increasingly saw how it helps to speak out and to be curious about others' perspectives instead of making assumptions, as well as bring difficult issues up for discussion.

The past year has felt like a rollercoaster. At times, with my fear of heights, it even felt like I was forced to jump from a great height. Uncomfortable but necessary to fly—or grow (Theme 5). By breaking out of my cocoon and embracing the unknown, I discovered new strength and joy in my work. I'm now more grounded and looking at my role from a fresh perspective.

**THEME 5:**
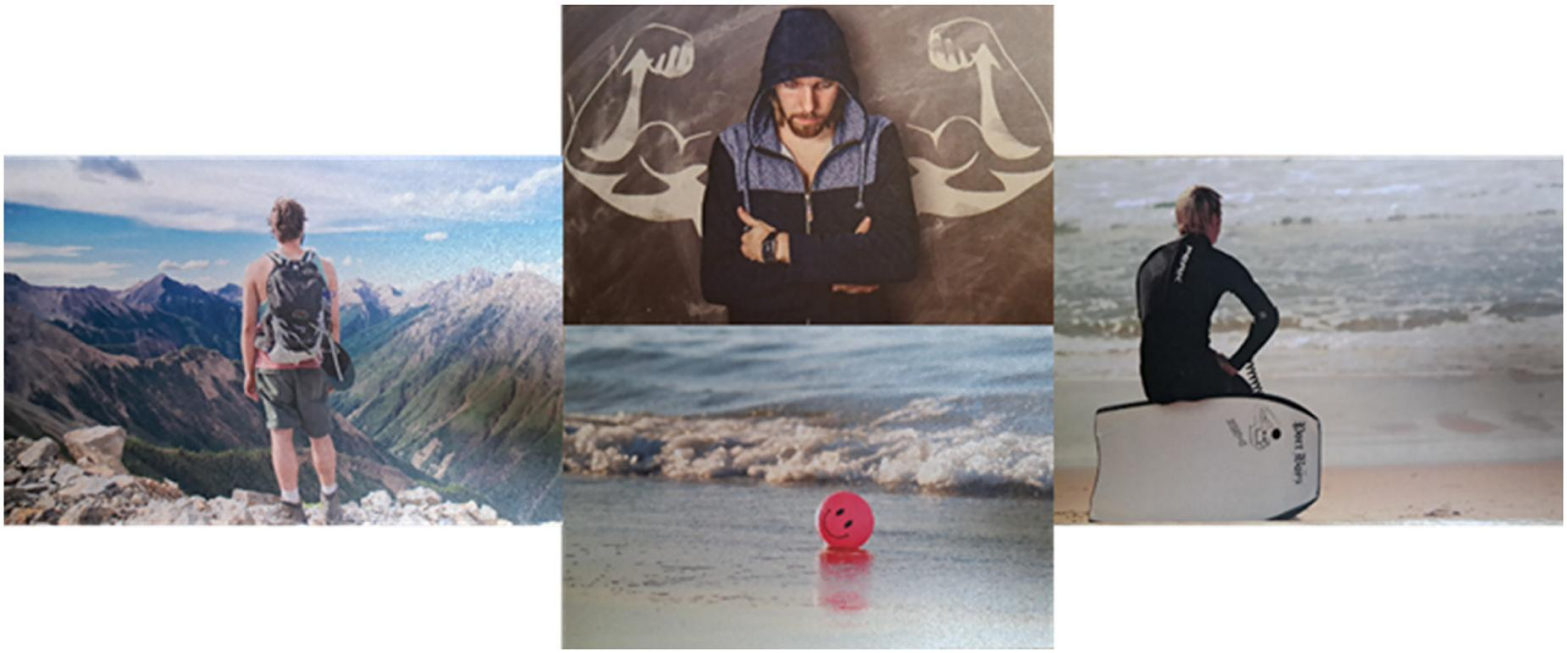
Dare to surf.

A key part of my leadership is building a safe environment. I work with a diverse team, and sometimes we encounter racism from residents. That's painful, but I've learned to engage in dialogue, explicitly support my staff, and make it clear that I stand by them. It's heartening to see that colleagues are now supporting and standing up for each other. I'm proud of that.

I learned that leadership isn't about a perfect script. Often, there are multiple options and no clear path. I had to take initiative myself. I spoke with people inside and outside the team, reviewed policy, puzzled over things, and searched for ways to create order, all whilst collaborating with staff. It became clear that change happens step by step: setting small goals, staying focused and taking new steps each time. There were certainly moments of doubt, wondering: “What am I doing?” But also moments of pride where I thought: “I've done this!”.

Another insight I've gained is the importance of celebrating successes. Not just the big milestones, especially the small moments too. A compliment, a successfully completed project, reflecting on what went well together—it helps people grow and makes me enjoy going to work more. This year has pushed me out of my comfort zone, sometimes against my will. But it has also given me focus and a new perspective. I've learned that perfection isn't achievable, that uncertainty is part of life, and that growth happens precisely by keeping moving. You only learn to surf by doing it!.

## Discussion

The case-narrative presented in this study is a qualitative thematised re-presentation of fourteen Belgian/Dutch front-line healthcare leader experiences as participants in the Leading to Connect and Retain (LCR) program of the Samen aan Z project (26). Quantitative evaluation and impact metrics are not available as the IT software developed for the “Samen aan Z” project proved to be too fragile for reliable data collection and analysis. However, coming from a person-centred perspective, we feel that the case narrative offers an inclusive impression of the participant experience as the diversity of individual experiences are captured within the five themes. Unique experiences have not been regarded as outliers or sided into the shadows, as occurs when the focus lies on the “average” experience.

The LCR program and development journey it supported was founded on principles of transformative and person-centred learning, as well as critical creativity. The use of creative methods in leadership development is relatively new but gaining popularity as educationalists/facilitators/coaches and researchers come to the realisation that leadership is more relational than rational and demands significant interpersonal (emotional and social) intelligence (40). Arts-based interventions aid perspective transformation as cognition connects with emotion, with the revelation and challenging of assumptions taking place as the relational and subjective aspects of leadership materialise. Garavan et al. (41) found that something as basic as a leadership drawing exercise can have a significant impact on participant emotional intelligence, leadership identity and feedback receptivity. During the LCR program, participant leaders were frequently invited to express their experiences using creative methods such as constructing a collage from art materials or using image cards to express their story. On the last day of the program, we intentionally invited participants to creatively express their developmental journey before they verbalised it, and so create the conditions for developmental narratives that are more cognitively and emotionally balanced. The fourth theme in the LCR case narrative reveals how critical creatively was valued for personal growth but it's adoption into the leaders' own (leadership) practice indicates the impact being transferred into organisations and teams. This is a valuable leadership skill in climates where team diversity can increase needs to help individuals find common ground and direction rather than remain entrenched in their own traditions and discourses. Creativity is increasingly being associated with effective leadership (development) (42) with creative leadership being described as the engagement of leader imagination to lead a group through a problem-solving process and find new directions for novel goals (43, 44). In transformational leadership, Kouzes & Posner (15) also recommends the critical practice of daring to question the status quo. Such leadership practice becomes critical when accompanied by leader reflexivity as the leader makes judgement-based decisions on how to act meaningfully. The case narrative is littered with expressions of how participants were learning to lead consciously, using creativity and criticality to co-create better places to work in.

Jack Mezirow, American sociologist and educator most frequently associated with transformative learning theory, states that deep, sustained learning occurs when adult learners unlearn outdated/non-useful habits, behaviours and beliefs, thereby freeing up space for more updated and useful ones. Consequently, learners become more inclusive, discriminating, open, reflective and emotionally capable of change (45). Ciporen (46) found that personally transformative learning has a positive, sustained impact on leadership style. The LCR case-narrative evidences such impact. Participants learned to “take conscious steps to help others discover their true colours and help them work together”, discarding their “limiting beliefs” and “consciously reflect on [their] choices”. “Giving them [staff] space and connecting with everyone's strengths” demonstrates how inclusivity influenced their leadership, and as they dared acknowledge their insecurities and accept making mistakes as part of growth, they became more emotionally equipped to “surf” the constant changes in their work context because “growth happens precisely by keeping moving”. Whilst there is evidence within the case narrative of participants fundamentally changing the way they view themselves as leaders and the environments they work in, the sustainability of these changes is not and could not be demonstrated in the case narrative. The data was collected at the end of the LCR program. In terms of the transformative process described by Brian Fay (33), consciousness-raising is evident as well as early signs of empowerment. Participant leaders were becoming aware of the need to change and the role they could play in creating workplace cultures where staff would want to stay. However, there is no indication that participants felt safe enough themselves within their current environment to continue their leadership development without the support from the program facilitators and learner community. This raises an important question that health and social care organisations could be asking themselves: how are we supporting continuous/can we support continued leadership development that is person-centred and transformative in nature?

The leadership theory and styles incorporated into the LCR program are collaborative and empowering in orientation. As facilitators of both cohorts, we often questioned whether participants themselves were experiencing collaborative and empowering leadership relationships themselves. Cave et al. (47) state that many nurse managers fail to receive organisational and upper management support for their leadership development. Their literature review concluded that mentor support from college managers or supervisors is important. Environmental constraints within the organisation was a frequent topic of dialogue and debate within the LCR sessions and both cohorts requested and positively evaluated a workshop exploring the exertion of influence horizontally and upwards. Constructive support was found within the community of learners on the LCR program, and such peer support should not be underestimated. Curry et al's (48) longitudinal evaluation of a leadership development program found that becoming a member of a professional network, with a common language and understanding, helps sustain participant embodiment and embeddedness of new ways of leading self and others. Debets et al's (49) realist evaluation of leadership development programs also concludes that when constructive feedback is fundamental to the program, participants become more self-aware and acquire insight into the needs and preferences of the people they lead and so exercise a more person-centred approach to leadership, which in turn benefits communication and collaboration within the workplace. Whilst these studies would suggest that similar outcomes could be expected among LCR participants, participant narratives were collected on the last day of the program, not 6–12 months later, so longitudinal changes to leadership (practice) could not be evidenced.

LCR participant learning can be viewed as person-centred, assuming that person-centred learning occurs when development is facilitated, not taught, and participant needs for safety, belongingness, self-esteem and freedom are respected and that reciprocal respect of personhood and mutual understanding emerges within learning relationships (32, 50, 51). The case narrative describes participants moving through discomfort to discover an authentic self as “the facilitators held up a mirror to me and asked questions that forced me to think more concretely and express my thoughts more clearly … and I increasingly saw how it helps to speak out and to be curious about others” perspectives instead of making assumptions'. Learning appears to have occurred in a safe environment where participants felt they belonged. The sharing of experiences, tips and tricks also gave them peace of mind and confirmation that they were not alone. Haber-Curran & Tillapaugh (52) also found that when leadership education is transformative and person-centred, participants experience a challenging of mental models, trust in the high challenge and high support offered by facilitators, freedom and empowerment to authentically and confidently take on challenges, as well as become more present and committed whilst reframing their beliefs about learning and the self as a leader of others.

Scheele (53) states that the chosen approach to leadership development is of importance if contemporary leaders are to become facilitators of transformative learning in practice, and guide staff to critically reflect on their practice in order to transform their ways of doing and being. Although not explicitly evidenced in the case narrative, the pedagogical foundations of the LCR program make it reasonable to expect that the program would foster transformational and person-centred leadership practices among participants. The peer-to-peer shadowing with focus group team discussion about the leadership they experienced as a team, was a learning activity offered within the program and would have generated evidence of leadership practice growth and development. Unfortunately, most participants did not take up the invitation to engage in peer-shadowing and team interviewing. We would, however, still support the inclusion of these activities within the LCR program as they have been shown to enhance collective reflection-in-action and responsive leader stancing (as described in person-centred leadership) (22, 54).

Among the wide range of positive outcomes described in Flaig et al's (55) review of leadership development, the LCR case narrative highlights the following outcomes: increased confidence and communication skills; job positivity and satisfaction; increased self-awareness; increased sense of a collective vision within the team and increased leader presence. These outcomes emerged over time, and continuous/longitudinal leader development is known to enhance leader resilience as leaders discover an authentic self and apply learning from a community of practice into their own workplace setting and leadership practice (56). However, Flaig et al. (55) are also clear that such beneficial outcomes are dependent on sustained participant attendance and engagement in the program. Of the 30 leaders who started the LCR program, only fourteen completed it. The case narrative presented here does not therefore include the experiences of sixteen participants who dropped-out along the way. The facilitator logbook does reveal that the most commonly reported reasons for drop-out were illness and heavy workload. What remains unknown is whether or not those who stopped prematurely experienced adequate support from within the workplace to enable continued participation in the program.

Edwards & Chargualaf (57) state that whilst there is no one best way to develop nurse leaders, active learning (including the four modes of dialogue with self and other, observation and action) and facilitation (appreciation of existing ability with high challenge and support to engage in new developmental activities) should be fundamental to the program. The LCR program's facilitated cooperative inquiry structure and process meets this recommendation. Hartviksen et al. (58) also state that leadership development programs, alongside sound pedagogical approaches, should focus on a transformative shift from solitary competitive leadership to collaborative networking, and from mission-based and controlling to empowering leadership. The theme “I don't have to be alone” illustrates this shift eloquently as team members are described as “stars” who need only direction and facilitation to embrace ownership and shared responsibility. There is also a realisation that support can be found from within the team, from “fellow managers” and from the “director”. This shows participants discovering supportive networks that extend beyond the LCR facilitators.

### Limitations

Whilst the case narrative presents a positive picture of transformative learning, there are some limitations that need to be highlighted. We (authors), as the programme facilitators, had noted in our own evaluations that participants often saw us as experts which limited their critical contestation of our underlying assumptions and the leadership theories we introduced them to. Whilst participants engaged in debate of their own assumptions and practical strategies, and we received feedback on our facilitation methods, there was minimal questioning of our core principles. This may have created social desirability effects, with unconscious adoption of our language and frames of reference which are then reflected in participant narratives. However, by the nature of the data gathering methods (creative expression followed by oral narration of the personal developmental journey), participants would only have shared what they felt was of importance to them.

Whilst participants were clearly engaged on a transformative journey, attrition and the moment of data gathering limit insight into (workplace) factors influencing engagement and development both during and after the program delivery. The perspectives of those who withdrew early from the program were not collected and as there was no post-program evaluations, sustained transformation of perceptions and leadership practice could not be evidenced. A follow-up invite for the fourteen participants to up-date their developmental narrative would be beneficial, preferably accompanied by observations and experiences of colleagues within the workplace.

Data triangulation with observations and experiences of those leading and being led by the LCR participants, would certainly have broadened the scope of impact evaluation. The aim of the LCR program was to facilitate discovery among leaders on how to cultivate workplace cultures, rather than to teach them specific leadership behaviours that should positively influence staff retention. However, failure of the project team to develop a functional app monitoring staff outcomes meant that we lack data supporting any assumptions that participant self-perceived transformation influences workplace environments and staff experiences. However, according to impact frameworks, changes in participant knowledge, attitudes, skills, relationships and behaviour represent the initial phase of individual and organizational change (Belcher & Halliwel). These initial changes are evident in the case narrative, but changes to the workplace culture and staff intent to stay are not evidenced.

With regards to program aims, McDonald (59) warns against making simplistic assumptions about the ability of improved leadership to solve complex problems such as healthcare staff retention. She also states that leadership development facilitators should be self-critical when designing, delivering and evaluating their program to ensure congruency between the explicit and implicit aims. The LCR program was designed by the authors of this paper, and the explicit intention was to support front-line leaders foster workplace cultures where healthcare professionals would want to continue to work. Cardiff et al's (12) four guiding “pillars” to support leader focus on what matters and justify their actions, was introduced in the program and is explicitly referenced in the case narrative. However, the case narrative does not include the healthcare staff voice, primarily because the shadowing and team focus group interviews did not take place at the start and end of the program. Even so, according to Belcher and Halliwell's (60) conceptualization of (research) impact, changes in the knowledge, attitudes, skills, relationships and/or behaviour of participants is the first phase of individual, organisational and systemic change. Such impact on participants is evident in the case narrative, but evidence for workplace culture transformation would require further data gathering.

## Conclusion

In an era when global healthcare is experiencing a recruitment and retention crisis within the largest professional workforce, organisational leaders are having to look at the role they and their leadership play in this wicked problem. Leadership development programs exist in abundance but are traditionally generic and academic in nature, or delivered locally with a specific focus. In both instances they tend to have predetermined structures and content focusing on specific leadership behaviours. This paper presents the evaluative findings of a leadership development program developed for a two-nation (Belgium and The Netherlands) project exploring the improvement of staff retention and organisational adaptability and innovative capacity within health- and social care organisations. The Leading to Connect and Retain program was a 12-month leadership development program based on transformative and person-centred learning principles. The program was delivered twice and on the last day participant front-line leaders creatively expressed then narrated their personal leadership journey. Fourteen personal narratives underwent a thematic analysis and the resultant framework used to construct a case narrative.

The case narrative offers insight into how frontline leaders in health and social care experienced the program aim of facilitating them in developing their adaptive capacity and agency within complex organizational environments. Their experience reflects the value of transformation and person-centredness in fostering authentic, meaningful growth as they embodied new ways of leading others and trying to embed new ways of working into their leadership practice. They describe emerging from a place of relative darkness into a space of colour where they can authentically lead others with increased confidence and build a stronger sense of shared responsibility. As designers and facilitators of the program, we (the authors) also feel that the cyclical co-operative inquiry process, critical and creative methods as well as responsive, person-centred facilitation, were key to participant transformation. Psychological safety within the learning community was an important antecedent to growth, we expect that respectful support and encouragement within the workplace will be of equal importance if sustainable and continued development is to be continued. The lack of staff outcomes during the program, as well as peer-shadowing data, has limited the scope of evidenced impact.

The participant case narrative illustrates how transformative leader development is not a linear process of familiarising self with pre-determined theories and methods, but an experimental journey requiring time, critical and creative reflection as well as person-centred support. Such conscious embodiment (authentically see & do) and embedding of person-centred and transformational ways of leading should cultivate healthful cultures where staff want to stay and work. Whilst the case narrative offers rich qualitative insights into participant transformation, more could be learned from longitudinal data gathering (during and after program completion) which incorporates multiple perspectives, particularly that of those being led by participant leaders.

## Recommendations

- Developers and facilitators of leadership development programs for front-line leaders in health and social care, should examine how transformative, person-centred and critical creativity principles can shape curriculum design and delivery.
- Funders, developers, facilitators and participants should pay explicit attention to post-program support (networks) and mentorship training, to enable continued leader and leadership growth and development.
- Sufficient time and support should be spent on motivating and enabling participant leaders to engage in peer-shadowing and gathering feedback data from peer-team members.
- Program evaluation and impact studies should include multiple voices, longitudinally and far into the post-program period in order to evidence change and impact. Particularly, staff outcomes such as intention-to-leave, job satisfaction, empowerment, belongingness, professional competency, leadership and vitality. whilst acknowledging the temporality of change in ecosystems.

## Data Availability

The raw data supporting the conclusions of this article will be made available by the authors, without undue reservation.
